# Passive eye movements induced by electromagnetic force (EMF) in rats

**DOI:** 10.24272/j.issn.2095-8137.2019.024

**Published:** 2019-05-18

**Authors:** Yue Yu, Jun Huang, Chun-Ming Zhang, Tian-Wen Chen, David S. Sandlin, Shao-Xun Wang, Alberto A. Arteaga, Jerome Allison, Yang Ou, Susan Warren, Paul May, Hong Zhu, Wu Zhou

**Affiliations:** 1 Departments of Otolaryngology and Communicative Sciences, University of Mississippi Medical Center, Jackson MS 39216, USA; 2 Department of Otolaryngology, First Affiliated Hospital, Shanxi Medical University, Taiyuan Shanxi 030001, China; 3 MD/PhD Program, University of Mississippi Medical Center, Jackson MS 39216, USA; 4 Pharmacology and Toxicology, University of Mississippi Medical Center, Jackson MS 39216, USA; 5 Neurobiology and Anatomical Sciences, University of Mississippi Medical Center, Jackson MS 39216, USA; 6 Neurology, University of Mississippi Medical Center, Jackson MS 39216, USA

**Keywords:** Eye movement, Proprioception, Extraocular muscles, Stretch reflex

## Abstract

Accurate information on eye position in the orbit is available from visual feedback, efference copy of the oculomotor commands and proprioceptive signals from the extraocular muscles (EOM). Whereas visual feedback and oculomotor commands have been extensively studied, central processing of EOM proprioceptive signals remains to be elucidated. A challenge to the field is to develop an approach to induce passive eye movements without physically contacting the eyes. A novel method was developed to generate passive eye movements in rats. A small rare-earth magnet disk (0.7 mm diameter, 0.5 mm thickness) was attached to the surface of a rat’s eyeball. A metal rod (5 mm diameter) wrapped with an electromagnetic (EM) coil was placed near the magnet (8–15 mm). By passing currents to the EM coil, electromagnetic force (EMF) was generated and acted upon the magnet and induced passive eye movements. The EMF induced well-defined passive eye movements, whose directions were dependent on current polarity and amplitudes and peak velocities were dependent on current intensity and duration. Peak velocities of the EMF-induced eye movements were linearly related to amplitudes, exhibiting main sequence relationships similar to that of saccades in awake rats and eye movements induced by electrical microstimulation of the abducens nucleus in anesthetized rats. Histological examination showed that repetitive EMF stimulations did not appear to result in damages in the EOM fibers. These results validated the EMF approach as a novel tool to investigate EOM proprioceptive signals and their roles in visual localization and gaze control.

## INTRODUCTION

An important issue in modeling visual localization and gaze control is to determine whether the central nervous system (CNS) obtains eye position information in the orbit via efference copy (or corollary discharge) of ocular motor commands or proprioception signals from the extraocular muscles (EOM) or the orbital connective tissues. Whereas Helmholtz (1925) emphasized the roles of efference copy, Sherrington (1918) emphasized the roles of EOM proprioception. The two camps have been debating this issue over the past century. On one hand, earlier studies showed that humans are aware of the passive displacements of the eyes in darkness (Skavenski, 1972) and altering this proprioceptive information consistently leads to errors in visual localization (Allin et al., 1996; Balslev & Miall, 2008; Bridgeman & Stark, 1991; Campos, 1986; Campos et al., 1989; Gauthier et al., 1990; Lennerstrand et al., 1997). Recent finding of EOM proprioceptive signals in the somatosensory cortex (Wang et al., 2007) and its roles in computing spatial maps (Xu et al., 2012) provided compelling evidence that the CNS utilizes the proprioceptive eye position signals. On the other hand, bilateral section of the monkey trigeminal nerves did not reduce accuracy of open-loop pointing (Lewis et al., 1994) and memory-guided saccades (Guthrie et al., 1983). Based on the assumption that the trigeminal nerves carry eye proprioceptive information to the brain (Porter et al., 1983), these results suggested that eye proprioception signal is not necessary for visual localization and gaze control because efference copy of the oculomotor motor commands is sufficient to implement these tasks.

The lack of understanding of the EOM proprioceptive signals is partially attributable to the fact that location of the peripheral sensors and their connections to the brain are still a matter of debate (Billig et al., 1997; Lienbacher et al., 2011a, 2011b; Zimmermann et al., 2011, 2013). However, it is also attributable to limitations of the three types of methods that have been used to induce passive eye movements. One method is to eliminate the EOM proprioceptive signals by use of surgical (Guthrie et al., 1983; Lewis et al., 1998) or pharmacological approaches (O’Keefe & Berkley, 1991). The second method is to mechanically displace an eye with a suction lens (Keller & Robinson, 1971) or by having the subject manually press on one of their eyes. The third method is to perturb the proprioceptive representation by vibrating the extraocular muscle tendon or electrically stimulating extraocular muscles (Han & Lennerstrand, 1999). These approaches suffer from the fact they may disrupt ongoing behaviors and create other sensory or motor signals. In the present study, we described a novel approach, which employs electromagnetic force (EMF) to induce passive eye movements in rats. The goal of the experiments was to demonstrate that the EMF approach is an effective tool for producing eye movements without inducing motor or non-proprioceptive somatosensory signals. Consequently, it can be used to determine the roles of eye position proprioceptive signals in visual localization and gaze control.

## MATERIALS AND METHODS

### Animals

A total of nine pigmented, female Long-Evans rats (Harlan Labs, Indianapolis, IN, USA) weighing 250–300 g were used in this study. Six of them were for EMF-induced eye movement experiments and three of them were for histological experiments. All procedures were carried out in accordance with NIH guidelines and approved by the Institutional Animal Care and Use Committee at the University of Mississippi Medical Center.

### Surgical procedures

**Figure 1:**
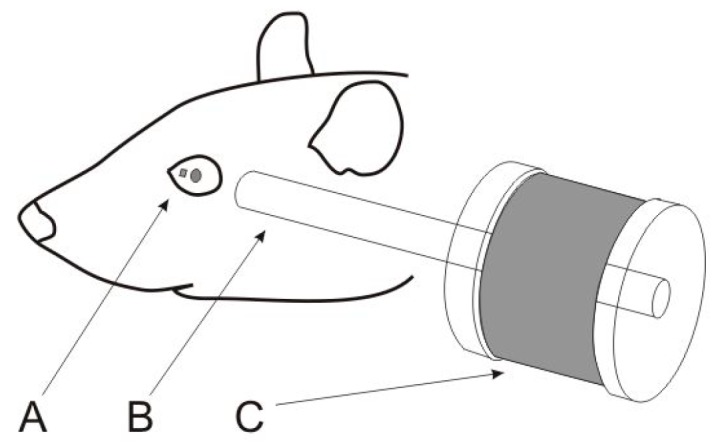
Schematic illustration of the experimenta

**Figure 2:**
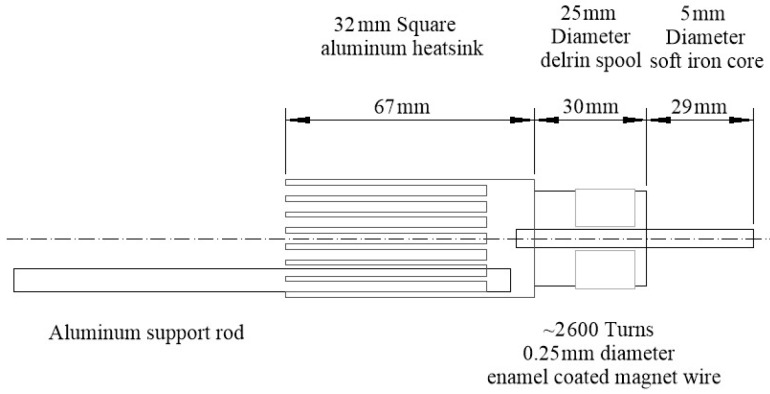
Schematic illustration of the electromagnetic device

All surgical procedures were performed aseptically, as described before (Zhu et al., 2011). Briefly, a rat was implanted with a small head holder on the skull and allowed 7 days for recovery before eye movement tests. On the day of testing, the rat was first anesthetized with 5% isoflurane-O_2_ (2 L/min). Once the rat was sedated, the isoflurane level was maintained at 2.5% throughout the rest of the procedure. A drop of ophthalmic solution (Proparacaine Hydrochloride Ophthalmic Solution, USP 0.5%, Valeant Pharmaceuticals North America LLC, NJ, USA) was applied on the top of corneal surface for local anesthesia. Next, a small magnetic disk ~0.7 mm in diameter and 0.5 mm in thickness was attached by 3 mol/L Vetbond (n-butyl-cyanoacrylate) tissue adhesive (St Paul, MN, USA) to the surface of the eyeball nasal to the pupil (Figure 1A). The line connecting the magnet poles was tangential to eye surface. A small soft iron core rod (5 mm diameter) was placed within 8–15 mm of the magnet and aligned with the line connecting the magnet poles. The end of the rod away from the magnet was inside of an electromagnetic coil (Figure 1, Figure 2), which generated magnetic field to magnetize the rod when current passed through the coil. The magnet field amplitude and onset were monitored by recording the EM coil current signal. The larger the activation current intensity (0.1–0.6 A), the stronger the electromagnetic field it generates. Based on the specifications of the EM coil (Figure 2), we used the following equation to estimate the magnetic flux (B) generated by activation current.

(1) B=n×I/(Lcore/μ+Lgap/μ0)

Where B is magnetic flux in Teslas, *n* is number of turns, I is activation current in Amperes, L_core_ is the length of the core in meters, L_gap_ is the distance from the core, μ is permeability of soft iron and μ_0_ is permeability of air. At 10 mm from the core, an activation current of 0.6 A generated a magnetic flux of about 0.2 Teslas.

After anesthesia, a small magnet was attached to the surface of the rat’s left eyeball nasal to the pupil (A). The line connecting the poles of the magnet was in the direction tangent to the eye surface. A small metal rod (B) was placed within 8–15 mm of the magnet and aligned with the line connecting its poles. The end of the metal rod away from the magnet was surrounded by an electromagnetic coil (C), which generated magnetic field to magnetize the metal rod when current passed through the coil.

It has a 59 mm soft iron core rod wrapped with ~2 600 turns of enamel coated magnet wire (0.25 mm diameter). The rod is attached to an aluminum heatsink and an aluminum support rod.

### Eye movement recording

Horizontal and vertical eye position signals were recorded using a video-based ISCAN ETS-200 eye tracking system (ISCAN, Burlington, MA, USA). An infrared camera equipped with a zoom lens (Computar Optics Group, Japan) was attached to the platform of the rotator/sled and was focused on the left eye of the rat, which was secured to platform via the head holder. The rat’s eye was illuminated by a standard ISCAN multiple infrared LED illuminator attached to the camera to produce a reference of corneal reflection (CR) for measuring eye position in the orbit. The eye tracker tracks the pupil center and the CR at a speed of 240 frames per second with a spatial resolution of 0.1 degrees. The differences of the two signals provided real-time signals related to eye position. Calibration was achieved by rotating the camera from left 10 degrees to right 10 degrees around the vertical axis of the recorded eye (de Jeu & de Zeeuw, 2012).

### Electrical stimulation of abducens nucleus

Rats were anesthetized by isoflurane with their heads fixed on the stereotaxic frame through the use of surgically implanted head holder. The right occipital bone was opened and the cerebellum exposed. A microelectrode pulled from a thin glass pipette (OD: 1.2 mm; ID: 0.9 mm; Sutter Instruments, Novato, CA, USA) was filled with 3 mol/L sodium chloride (10–20 MΩ) and was advanced to the abducens nucleus using coordinates in the atlas of Paxinos & Watson (2009). The abducens nucleus was identified as the site where abducting eye movements were evoked by low intensity electrical stimulation (10 mA, 200 ms duration, 100 Hz, pulse duration 0.2 ms) delivered through the recording electrode. Stimulation-evoked eye movement was recorded by the eye tracker. By varying current intensity, electrical stimulation evoked eye movements with varying amplitudes were produced. After completion of the experiments, the recording sites were marked by ejecting fast green with a negative 7 mA current for 10 min, and their locations were subsequently verified histologically.

### Data acquisition and analysis

Signals related to horizontal and vertical eye position and detecting coil voltage were sampled at 1 kHz at 16 bits resolution by a CED Power 1401 system (Cambridge Electronics Devices, Cambridge, UK). Eye movement responses were analyzed using Spike2 (Cambridge Electronics Devices, Cambridge, UK). Raw eye position data were filtered and differentiated with a band-pass of DC to 50 Hz to obtain eye velocity data. Trials in which there was no pupil tracking within 50 ms of the onset of the EMF onset were rejected (less than 5%). Trials in the data stream were aligned with the EMF onset and averaged (~100 trials per condition) to obtain low-noise estimates of eye position and velocity as a function of time. The EMF induced a displacement of eye position, whose direction was dependent on the EMF polarity (attractive or repulsive). After the EMF was turned off, the eye returned to its initial position with an exponential time course, which was fitted by a sum of two exponentials (Anderson et al., 2009).

(2) $$A(t)=A_{1}e^{-(t/T1)}+A_{2}e^{-(t/T2)}$$

### Examination of extraocular muscle morphology

In order to determine whether repetitive EMF-induced movements of the eye damaged the orbital contents, the eyes of three rats were examined histologically. Following completion of >3 000 EMF-induced eye movements toward the ear (i.e., stretching the medial rectus), rats were sacrificed via intracardiac perfusion with 4% paraformaldehyde in pH7.2, 0.1 mol/L phosphate-buffered saline (PBS). The orbit and its contents (bone, globe, extraocular muscles and glands) were harvested. The globe and extraocular muscles were placed in 30% sucrose in pH 7.2, 0.1 mol/L phosphate buffer (PB) solution. They were then cryoprotected by moving them through increasingly concentrated solutions of Cryomatrix embedding medium (Shandon Cryomatrix, Thermo Fisher Scientific Inc., DE, USA). The globe was then oriented in a disposable mold containing Cryomatrix and frozen. Once the specimen was cooled to −19 °C, individual sections containing the globe and extraocular muscles were cut serially at 35 µm on a cryostat (Thermo Shandon Cryotome E cryostat, Thermo Fisher Scientific Inc., DE, USA). Sections were secured to cold, gelatin coated glass slides. They were then stained using Masson Trichrome stain, cleared and cover slipped. Digital photographs were taken using NIS-Elements AR and a Nikon Eclipse E600 light microscope equipped with a 1.5 megapixel Nikon DS-Ri1 high resolution camera. When necessary, images were adjusted for brightness, contrast and color using Adobe Photoshop CS5 to replicate the image as it appeared when visualized under the microscope.

## RESULTS

### Passive eye movement induced by EMF

**Figure 3:**
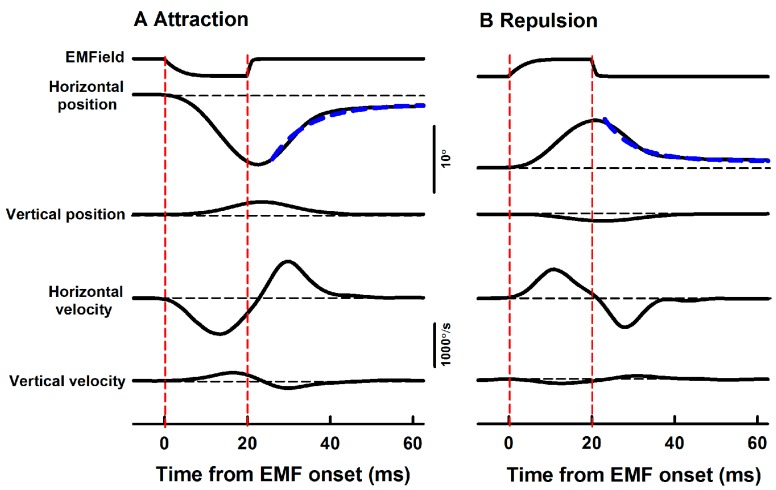
Averaged eye position and velocity responses to EMF (Rat1)

The EMF induced well-defined eye movements in both horizontal and vertical directions. In the example shown in Figure 3, negative current pulses (20 ms, −0.55 A) generated attractive EMF to the magnet at a distance of 10 mm, which rotated the left eye toward the left ear with a latency of ~5 ms, resulting in a passive eye movement of 11.4 deg in the leftward horizontal direction and 2.2 deg in the upward direction (Figure 3A). About 11 ms after the EMF was turned off, the elasticity of the eye plant drove the eye toward its initial position with exponential a time course. Plots of this movement could be fitted with two time constants (8.5 ms and 557 ms, R^2^=0.99, Figure 3A, blue dotted line). Positive current pulses (20 ms, 0.55 A) generated repulsive EMF to the magnet, which rotated the left eye away from the left ear with a latency of ~5 ms, resulting in a passive eye movement of 7.4 deg in the rightward horizontal direction and 1.0 deg in the downward direction (Figure 3B). About 9 ms after the EMF was turned off, the elasticity of the eye plant drove the eye back to its initial position with an exponential time course, which could be fitted with two time constants (6.7 ms and 228.1 ms, R^2^=0.99, Figure 3B, blue dotted line).

A: Attractive EMF induced passive eye movements of the left eye toward the left ear. B: Repulsive EMF induced passive eye movements of the left eye away from the left ear. The vertical dashed lines indicate EMF onset (current pulse: 0.55 A; 20 ms). Horizontal dashed lines indicate initial eye position and baseline eye velocity (zero). Upward traces indicate eye movements away from the left ear. Downward traces indicate eye movements toward the left ear.

**Figure 4:**
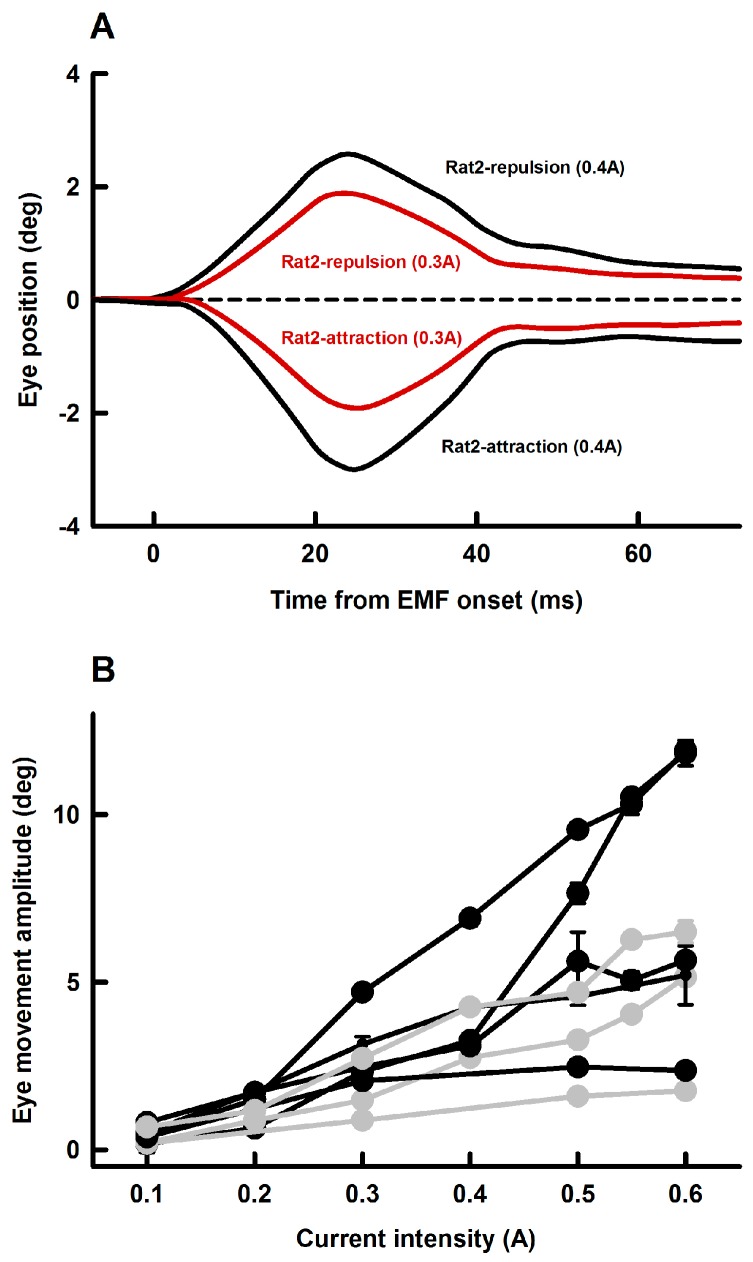
Effect of EM coil current intensity on EMF-induced eye movements

To assess the effectiveness of using EMF to induce passive eye movements, we examined effects of EM coil current intensity (0.1 A–0.6 A) on amplitudes of EMF-induced eye movement. Figure 4A shows representative eye responses (Rat2) at two EM coil current intensities (0.3 A and 0.4 A) and two polarities (repulsion and retraction) at a distance of 10 mm. EM coil currents with higher intensities induced eye movements with larger amplitudes. Figure 3B summarizes eye movement responses from four rats with attraction EMF (black symbols and lines) and three rats with repulsion EMF (grey symbols and lines). Eye movement amplitudes increased linearly with EM coil intensity. For the four cases with attraction EMF (Figure 4B, black lines), the slopes of eye movement-current intensity are 23.6±0.9 deg/A, 24.3±3.6 deg/A, 10.2±1.1 deg/A and 3.9±1.4 deg/A, respectively. For the three cases with repulsion EMF (Figure 4B, grey lines), the slopes of eye movement-current intensity are 12.2±0.9 deg/A, 9.4±0.8 deg/A and 3.2±0.2 deg/A, respectively.

A: Representative averaged eye movement responses of Rat2 at two current intensities for the two polarities. Error bars indicating standard error are within the thickness of the lines. B: Summary of amplitudes of EMF-induced eye movements as functions of current intensity from 4 rats with attraction EMF (black symbols and lines) and 3 rats with repulsion EMF (grey symbols and lines). EM distance from the magnet on the rat eye (10 mm) and EM duration (20 ms) remained unchanged throughout the measurements. Error bars indicating standard errors are within the thickness of the symbols.

**Figure 5:**
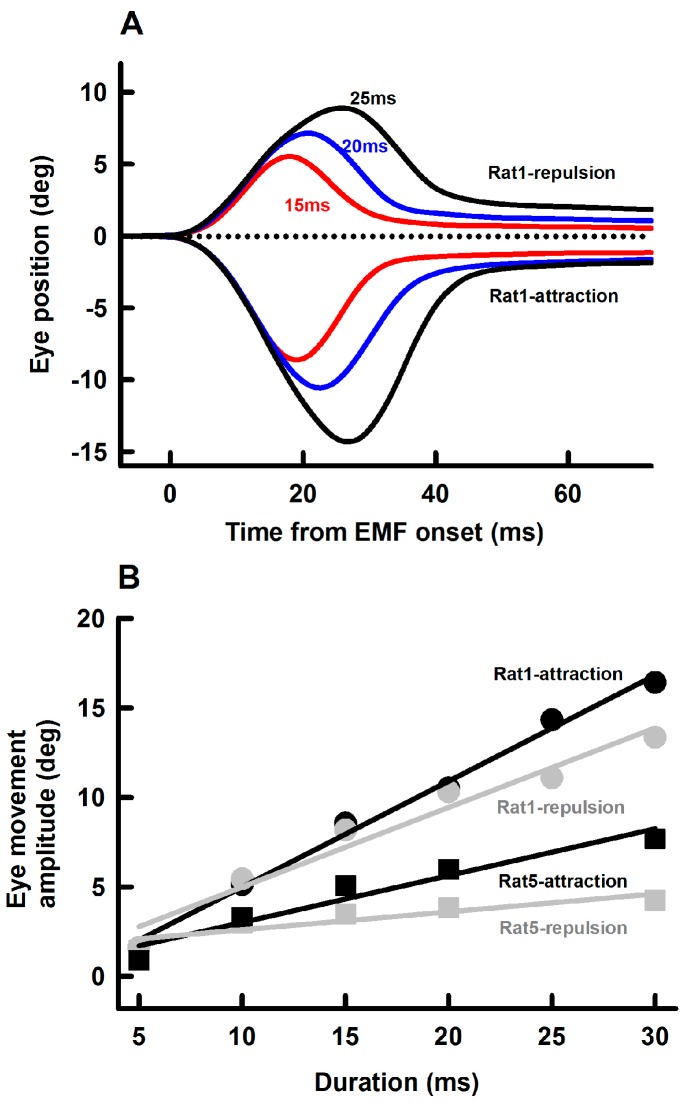
Effect of EM current duration on EMF-induced eye movements

We also examined effects of EM coil current duration (5–30 ms) on amplitudes of the EMF-induced eye movement. Figure 5A shows representative eye responses at three EM coil current durations (15 ms, 20 ms and 25 ms, 0.55 A intensity) and two polarities (repulsion and retraction) at a distance of 10 mm. EM coil currents with longer durations induced eye movements with larger amplitudes. Figure 5B summarizes eye movement responses from 2 rats with attraction EMF (black symbols and lines) and repulsion EMF (grey symbols and lines). Eye movement amplitude increased linearly with EM coil current duration. For attraction EMF, the slopes of eye movement-current duration are 0.59±0.03 deg/ms and 0.26±0.04 deg/ms for Rat1 and Rat5, respectively. For repulsion EMF, the slopes of eye movement-current duration are 0.44±0.05 deg/ms and 0.1±0.03 deg/ms for Rat1 and Rat5, respectively.

A: Representative averaged eye movement responses (Rat1) at three current durations for the two polarities. Error bars indicating standard error are within the thickness of the lines. B: Amplitudes of EMF-induced eye movements as functions of current duration from Rat1 and Rat5. Other parameters (EM distance from the magnet on the rat eye: 10 mm; EM current: 0.55 A) remained unchanged throughout the measurements. Error bars indicating standard errors are within the symbols.

### Effects of the magnet-metal rod distance on the EMF-induced eye movement

**Figure 6:**
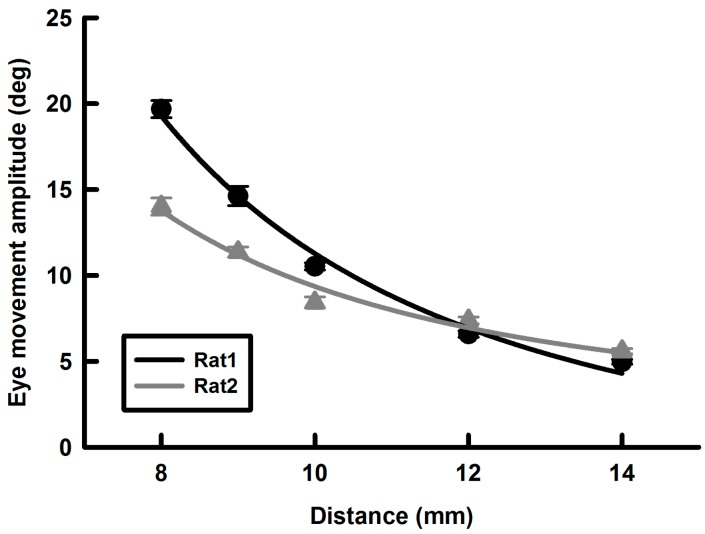
Effect of the distance between the tip of the metal rod (C in Figure 1) and the magnet on the rat eye (B in Figure 1) on EMF-induced eye movements

Since the EMF is inversely related to square of distance between the magnet and the magnetized metal rod, the amplitudes of the EMF-induced eye movements were expected to be inversely related to square of the distance. Figure 6 plotted the amplitudes of EMF-induced eye movements as a function of the distance for two rats. The two solid lines show that the following equations provide excellent fits of the data.

(3) $$\begin{array}{c} E=1424.9/D^{2}\\ \;\;\;(R^{2}=0.99,\mathrm{Rat}1) \end{array}$$

(4) $$\begin{array}{c} E=786.7/D^{2}\\ \;\;\;\;\;(R^{2}=0.99,\mathrm{Rat}2) \end{array}$$

Where E is the amplitude of EMF-induced eye movement and D is the distance between the magnet and the metal rod (Figure 1).

Red dots are for Rat1 and blue dots are for Rat2. EM current was 0.55 A and EM duration was 20 ms. Error bars indicate standard errors are within the symbols.

### Main sequence of EMF-induced eye movement

**Figure 7:**
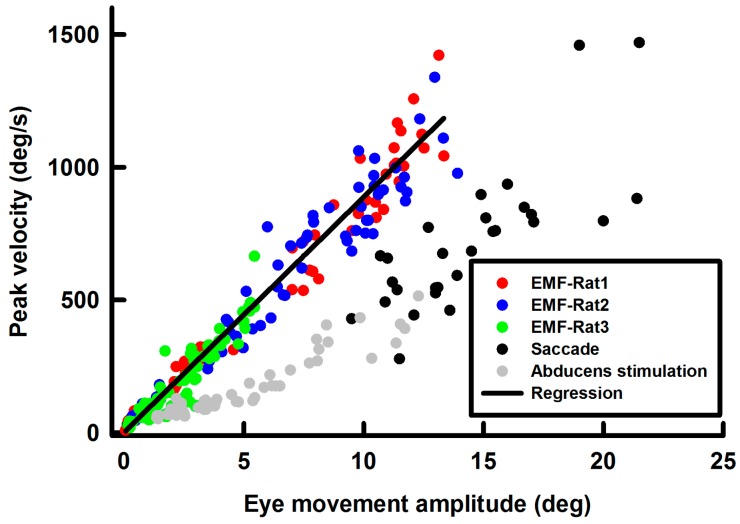
Main sequence of EMF-induced eye movements (each rat is indicated by different colored symbols)

To assess the dynamics of the EMF-induced eye movements, we compared their main sequences to that of saccades in awake rats and the eye movements evoked by microstimulation of the abducens nucleus in anesthetized rats. Peak velocities of the EMF-induced eye movement were plotted against their amplitudes for three rats (Figure 7, green/blue/red symbols). Data from the three animals fell on a single straight line with a slope of 89±1 deg/s/deg (R^2^=0.98), indicating a well-defined main sequence for this condition. However, since EMF had a much more rapid onset than the muscle forces generated during saccade and abducens nucleus stimulation, the main sequence of the EMF-induced eye movements exhibited a steeper slope than that of the awake saccade (Figure 7, black symbols, slope of 65±10 deg/s/deg, R^2^=0.62), as well as eye movements induced by abducens nucleus stimulation in anesthetized rats (Figure 7, grey symbols, slope of 36±2 deg/s/deg, R^2^=0.9) (*P*<0.01).

The grey symbols and the black symbols are for abducens stimulation-induced eye movements in anesthetized rats and saccades in awake rats, respectively.

### Effects of passive stretching on the extraocular muscle morphology

**Figure 8:**
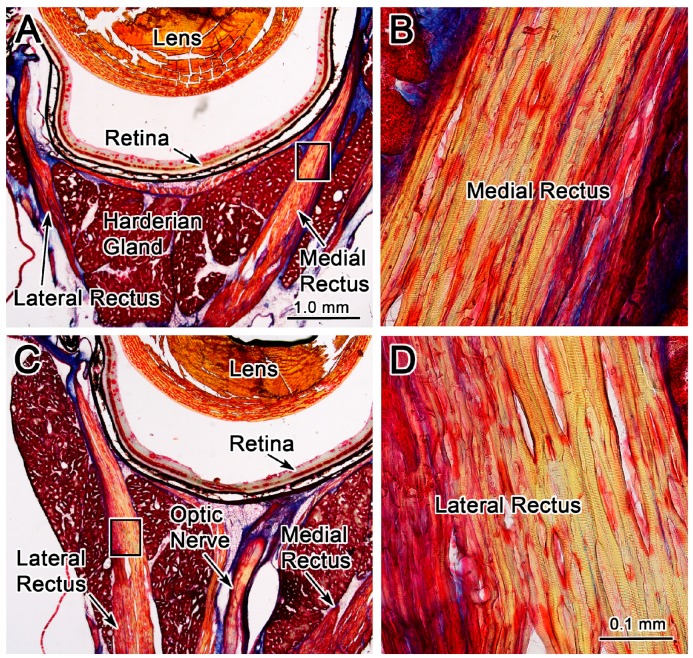
Morphologic evidence against EMF-induced damage in extraocular muscle fibers

The eyes used in these experiments were stained with Masson trichrome stain to evaluate whether repeated FMF-induced eye movements resulted in morphological changes. Since the EMF-induced eye movements were always in the abduction direction for this experiment, any strain was presumed to be induced on the medial rectus muscle, which was stretched without the tonic eye position motor commands being suppressed. The lateral rectus, would be shortened under these procedures. Figure 8 shows photomicrographs of a section taken through a left globe containing both the stretched medial rectus muscle (Figure 8A, B) and the compressed lateral rectus muscle (Figure 8C, D). The EMF-induced eye movements did not appear to displace the medial rectus muscle from its normal location between lobes of the Harderian gland (Figure 8A). The medial rectus muscle scleral insertion appeared intact. No obvious tearing or separation of the muscle insertion were noted. The morphology of the neurovascular bundles (not illustrated) in the stretched medial rectus muscle was similar to the control lateral rectus muscle. Medial rectus and lateral rectus muscle structure (boxes in Figure 8A, C) are shown at high magnification (Figure 8B, D). The stain revealed similar striation patterns for the medial rectus (Figure 8B) and lateral rectus (Figure 8D) muscles. No obvious structural changes were seen when viewed at higher magnification. Taken together, the medial rectus muscle morphology, as determined by this qualitative assessment, appeared unchanged by this perturbation. We hypothesized that damage to the medial rectus muscle structure might manifest as increases in local inflammatory response including an increase in inflammatory cells or extravasation of blood into the surrounding connective tissue. However, no obvious increase in the inflammatory cells or accumulation of blood within the muscle or surrounding connective tissue was observed in the orbit. Thus, based on the qualitative light microscopic analysis, the EMF-induced eye movements observed in this study did not appear to produce gross changes to the EOM morphology.

Photomicrographs of Mason trichrome stained sections through the left globe at the level of the medial rectus (stretched) and the lateral rectus (compressed) muscles. Low power photomicrographs show the organization of the medial rectus (A) and lateral rectus (C) muscles. When the medial rectus muscle (box in A) and the lateral rectus muscle (box in C) are viewed at higher magnification in B and D respectively, they appear the same. Scale bars: 1.0 mm for A and C; 0.1 mm for B and D.

## DISCUSSION

The feasibility of using EMF to generate eye movement was first demonstrated by Bohlen & Chen (2016) in a ping-pong ball model. In the present study, we presented further evidence in a rat model that the EMF induced well-defined passive eye movements over a range of amplitudes (up to 15 deg) and peak velocities (up to ~1 500 deg/s) without causing damages to the extraocular muscles. The EMF approach offers important features that make it an excellent tool to study neural processing of EOM proprioception signals in awake animal models. First, the EMF generates passive eye movement without physically contacting the eye. This feature is essential for perturbing fixation or ongoing eye movements in awake and behaving subjects. Second, the EMF induces eye movement with a very short latency (<5 ms). Since a typical saccadic eye movement lasts only 20–50 ms, the rapid activation and deactivation of the electromagnet minimizes potential biases of subjects’ attention and anticipation. Third, by selecting appropriate current parameters (polarity, intensity and duration), passive eye movements can be generated with desired direction, amplitude and duration. This feature allows generation of a wide range of passive movements to fully characterize neural encoding of the EOM proprioceptive signals. Fourth, by varying magnet and rod positions, it is possible to perturb different EOMs and generate passive eye movements in different directions. This would overcome the limitations of the vibratory method, which primarily activates the inferior rectus muscle, as the tendons of other EOMs are difficult to reach via this technique (Han & Lennerstrand, 1999; Velay et al., 1997).

### Previous studies on proprioceptive signals of the EOMs

There is a long history of eye muscle proprioception investigation (for review see Donaldson, 2000). Since it is difficult to access the primary trigeminal afferent neurons under chronic conditions, early studies of extraocular proprioceptors primarily relied on examining the effects of sectioning the ophthalmic branch of the trigeminal nerve (Guthrie et al., 1983; Lewis et al., 1998) or surgically altering EOM insertions to alter the position of an eye in the orbit (Steinbach & Smith, 1981). Although important insights have been gained in these studies, their approaches were invasive, irreversible, and the results may have been confounded by central nervous system compensation. Investigators have also tried to directly manipulate proprioceptive sensory signals while subjects performing visuo-motor tasks, by pulling on the eye via sutures or suction, or by tugging or vibrating eye muscles. For example, Keller & Robinson (1971) trained monkeys to fixate a visual target with one eye while the other eye was passively pulled by force applied to a contact lens. Abducens neurons on the side of the pulled eye were recorded. They found that the abducens neuron activity exhibited no change in response to the passively generated eye movements, suggesting that there was no stretch reflex in the lateral rectus muscle. While these results were widely interpreted as evidence against a role of the EOM afferent signals in eye movement control, it is important to note the study’s limitation, i.e., it only generated slow passive eye movements and the eye was not moving. It did not rule out the possibility that stretch reflexes may be present if the eye was moving at high velocity. Indeed, another study using contact lenses to impede the movements of an eye during saccades showed changes in the contralateral eye (Knox et al., 2000). Furthermore, in a recent study in anesthetized rats and squirrel monkeys, Dancause et al. (2007) manually rotated the eye, while measuring the EMG activity in the lateral rectus muscle. By producing passive eye movements of ~25 deg within about 100 ms (eye velocity of 200 deg/s), they observed typical stretch reflexes in these muscles.

In addition to using surgical and mechanical approaches to manipulate the proprioceptive EOM signal, pharmacological approaches have also been used to investigate the functional roles of EOM proprioceptive signals. For example, O’Keefe & Berkley (1991) injected dibucaine (a high-potency paralytic agent) in one eye of anesthetized cats to block sensory nerve transmission in the eye. They found that the injection reduced frequency and amplitude of spontaneous eye movement in both the treated and non-treated eyes and concluded that proprioceptive sensory signals from one eye are used to modulate movements of the other eye. This is consistent with the fact that the unilateral trigeminal inputs are distributed bilaterally by trigemino-trigeminal projections (Warren & May, 2013).

In contrast to the extensive studies on the behavioral effects of manipulating EOM proprioceptive signals, few studies have examined the neural substrates of the EOM proprioceptive signals. In an earlier study, Fuchs & Kornhuber (1969) reported evoked potential responses in cat cerebellum while their eyes being rotated at saccade velocities. In an important recent study, Goldberg and colleagues discovered a representation of proprioceptive eye position signals in the somatosensory cortex of behaving monkeys (Wang et al., 2007). To elucidate the nature of these eye position-related signals, they injected lidocaine into the retro-orbital space of an eye in awake behaving monkeys to block all sensory inputs from the eye. The injection substantially reduced eye position-sensitive neuronal activity in the somatosensory cortex. Their subsequent studies of gain fields in parietal cortex suggest that while corollary discharge is used for guiding the initial saccades in a memory guided series, proprioception plays the more important role in subsequent saccades in the series (Sun & Goldberg, 2016; Xu et al., 2012).

### Future studies

In comparison with neural processing of corollary discharge signals related to oculomotor commands (Sommer & Wurtz, 2008), neural processing of EOM proprioceptive signals has received much less attention. However, understanding the EOM feedback signals and their interactions with the feedforward efference copy signals has important functional and clinical implications. It was the finding of EOM proprioceptive signals in the somatosensory cortex (Wang et al., 2007) that reignited interest in elucidating the role that EOM proprioceptive signals play for gaze control and spatial perception at different levels of the oculomotor system. The present study was an effort to develop the EMF approach for effective manipulation of proprioceptive signals without changing ongoing motor commands. The results demonstrate the feasibility of using EMF approach in other species, including monkeys. Ongoing studies are directed at improving the EMF approach in two ways. One is to develop a ferrous metal ring that can be implanted under the conjunctiva of the eyes. The other one is to further develop the EM coil and control of the activation current so that both we can either generate an eye movement with desired speeds and amplitudes or hold the eye at any desired position for a period of time. This approach will offer an important technical advancement to facilitate behavioral and neurophysiological studies in awake and behaving subjects.
